# Evaluation of Bone Mineral Density and Related Factors in Romanian HIV-Positive Patients Undergoing Antiretroviral Therapy

**DOI:** 10.3390/microorganisms13081768

**Published:** 2025-07-29

**Authors:** Ioana-Melinda Luput-Andrica, Adelina-Raluca Marinescu, Talida Georgiana Cut, Alexandra Herlo, Lucian-Flavius Herlo, Andra-Elena Saizu, Ruxandra Laza, Anca Lustrea, Andreea-Cristina Floruncut, Adina Chisalita, Narcisa Nicolescu, Cristian Iulian Oancea, Diana Manolescu, Romanita Jumanca, Daniela-Ica Rosoha, Voichita Elena Lazureanu

**Affiliations:** 1 Doctoral School, Victor Babes University of Medicine and Pharmacy Timisoara, E. Murgu Square, Nr. 2, 300041 Timisoara, Romania; ioana.luput-andrica@umft.ro (I.-M.L.-A.); flavius.herlo@umft.ro (L.-F.H.); andra.saizu@umft.ro (A.-E.S.); andreea.floruncut@umft.ro (A.-C.F.); adina.chisalita@umft.ro (A.C.); daniela.rosoha@umft.ro (D.-I.R.); 2 Department XIII, Discipline of Infectious Diseases, Victor Babes University of Medicine and Pharmacy Timisoara, E. Murgu Square, Nr. 2, 300041 Timisoara, Romania; talida.cut@umft.ro (T.G.C.); alexandra.mocanu@umft.ro (A.H.); laza.ruxandra@umft.ro (R.L.); nicolescu.narcisa@umft.ro (N.N.); lazureanu.voichita@umft.ro (V.E.L.); 3 Center for Ethics in Human Genetic Identification, Victor Babes University of Medicine and Pharmacy Timisoara, E. Murgu Square, Nr. 2, 300041 Timisoara, Romania; 4 Department of Educational Sciences, University Clinic of Therapies and Psycho-Pedagogical Counseling, West University of Timisoara, 300223 Timisoara, Romania; anca.lustrea@e-uvt.ro; 5 Department XIII, Discipline of Pneumology, Victor Babes University of Medicine and Pharmacy Timisoara, E. Murgu Square, Nr. 2, 300041 Timisoara, Romania; oancea@umft.ro; 6 Center for Research and Innovation in Precision Medicine of Respiratory Diseases (CRIPMRD), Victor Babes University of Medicine and Pharmacy Timisoara, E. Murgu Square, Nr. 2, 300041 Timisoara, Romania; 7 Department XV, Discipline of Radiology, Victor Babes University of Medicine and Pharmacy Timisoara, E. Murgu Square, Nr. 2, 300041 Timisoara, Romania; diana.manolescu@umft.ro; 8 Romanian and Foreign Languages Department, Victor Babes University of Medicine and Pharmacy Timisoara, 300041 Timisoara, Romania; romanita.jumanca@umft.ro

**Keywords:** HIV, AIDS, PLWH, bone mineral density, antiretroviral therapy, smoking, osteopenia, osteoporosis, risk factors

## Abstract

Human Immunodeficiency Virus (HIV) infection remains a major global health issue, with effective antiretroviral therapy (ART) extending life expectancy but also increasing age-related issues like osteopenia and osteoporosis. This cross-sectional study examines bone mineral density (BMD) and related risk factors in Romanian HIV-positive patients, emphasizing regional and therapy influences. The patients varying in HIV infection duration underwent DXA scanning to measure BMD in the lumbar spine, femoral neck, and total femur. A high prevalence of low BMD, especially in the lumbar spine, was identified along with significant associations between reduced BMD and factors such as smoking, alcohol use, vitamin D deficiency and serum phosphorus levels. ART like Protease Inhibitors and Nucleoside Reverse Transcriptase Inhibitors were linked to increased bone loss, emphasizing the multifactorial nature of osteoporosis in HIV-infected individuals and underscore the importance of regular BMD assessments, lifestyle adjustments, and careful management of antiretroviral therapy to minimize fracture risk and enhance overall health and quality of life.

## 1. Introduction

The Human Immunodeficiency Virus (HIV) continues to represent a major global public health concern, affecting millions worldwide and contributing to substantial morbidity and mortality across various regions [1]. By the end of 2023, approximately 39.9 million people were living with HIV (PLWH) globally, including 38.6 million adults [34.9–43.1 million aged 15 and above] and 1.4 million children [1.1–1.7 million aged 0–14 years] [2]. Women and girls accounted for 53% of all individuals living with HIV [2]. In the same year, worldwide, about 1.2 million adults acquired HIV globally [950,000–1.5 million].

In Europe, an estimated 3.1 million people were living with HIV, with only about 62% [49–71%] receiving antiretroviral therapy. During this period, around 160,000 individuals contacted the virus [0–48,50–190], marking an incidence rate increase from 0.17 to 0.18 per 1000 uninfected individuals since 2010. HIV-related deaths reached an estimated 49,000, representing a 27% rise since 2010, highlighting the ongoing impact of the epidemic [3,4]

HIV infection significantly impairs patients’ health, potentially progressing to AIDS—a severe stage marked by extreme immune suppression and heightened susceptibility to opportunistic infections and related conditions [5]. Among common complications is the loss of BMD [6], which can lead to osteopenia and osteoporosis. The likelihood of developing osteoporosis escalates markedly with age, increasing by 1.4 to 1.8 times per decade beyond 30 years of age [7,8].

Bone imbalances in HIV-positive patients can be attributed to a series of cumulative factors. The infection itself triggers the chronic inflammatory response, with the release of proinflammatory cytokines that negatively affect bone formation. Additionally, certain classes of antiretroviral medications, particularly protease inhibitors (PI) [9,10,11] and tenofovir disoproxil fumarate(TDF) [12], have been associated with altered bone turnover and an increased fracture risk. Shared risk factors linked to decreased bone density in individuals with HIV include low body weight [8] over 50 years of age [8], reduced CD4 cell count [13], inadequate calcium and vitamin D intake, sedentary lifestyle [14], smoking [15], excessive alcohol consumption, and the virus’s direct impact on bone metabolism [16,17].

According to the Romanian National Institute of Public Health, as of December 31, 2024, the number of PLWH/AIDS in Romania was 18,768. Of these, 141 were children aged 0–14 years, 133 were adolescents aged 15–19 years, and 18,494 were adults aged 20 years or older [18]. The primary transmission modes for newly reported cases were unprotected heterosexual intercourse (60% of cases), unprotected sexual contact between men who have sex with men (MSM) (31%), and injection drug use (7%) [19].

In the current scenario, characterized by increased longevity among HIV-positive individuals due to the effectiveness of ART, evaluating chronic complications associated with HIV infection and its treatment is crucial. One such complication is the alteration of bone metabolism, resulting in decreased BMD [20], which may lead to osteopenia and osteoporosis, especially among middle-aged and elderly patients [21,22].

Although data on osteoporosis prevalence in Romanian individuals living with HIV are limited [19], this cross-sectional study aims to assess BMD in HIV patients, targeting the identification of regional risk factors contributing to bone imbalances and exploring potential preventive strategies. The increased life expectancy of HIV-infected individuals, fueled by effective antiretroviral therapies, has led to a higher occurrence of age-related complications, such as senile osteoporosis [23,24], significantly impacting quality of life and increasing fracture risks.

## 2. Materials and Methods

### 2.1. Study Design and Participants

This research was designed as a cross-sectional study aimed at evaluating BMD among PLWH. The sample size was determined based on a preliminary statistical analysis, using a one-tailed z-test for a large sample and an estimated odds ratio for lumbar and left lower limb (hip and femur) bone density, according to data from previously studied Romanian HIV-positive populations [25,26,27]. Calculations targeted a statistical power of 95% with a significance level of 0.05. Participants were recruited via convenience sampling from approximately 550 patients actively registered at the HIV Clinic of the “Dr. Victor Babes”, Clinical Hospital for Infectious Diseases in Timisoara.

Exclusion criteria were meticulously defined to minimize potential confounders affecting BMD values and included the following: patients experiencing menopause (either physiological or surgical), individuals with congenital or acquired bone disorders (osteogenesis imperfecta, osteomalacia, Paget’s disease), endocrine disorders (hypogonadism, hyperthyroidism, hyperparathyroidism), type 1 and 2 diabetes mellitus, a history of malignancy, inflammatory joint diseases (rheumatoid arthritis, spondylarthritis, psoriatic arthritis), neuromuscular conditions (myopathies, muscular dystrophy, myasthenia gravis), moderate to severe chronic kidney diseases (eGFR < 60 mL/min/1.73 m^2^), prolonged immobilization, recent exposure to iodinated contract substances (within 72 h prior to the DEX assessment), presence of metallic implants, orthopedic prostheses, or cardiac pacemakers (contraindications for DXA), body weight > 136 kg, or height > 195 cm (technical limits for a DXA machine), as well as inability to maintain a still position during the scan. Pregnant women and those already undergoing treatment for osteoporosis were also excluded.

Ultimately, 163 patients were included, comprising 106 men and 57 women, aged between 18 and 80 years old, all diagnosed with HIV and on a stable antiretroviral therapy regimen for at least six months, indicating uninterrupted use of the same combination of HIV medications during this period. Evaluations were conducted from 2023 to 2025 and provided informed consent for undergoing dual-energy X-Ray absorptiometry scanning, used to determine BMD.

### 2.2. Study Measurement

Participants were recruited from among patients undergoing active antiretroviral therapy and registered at the “Dr. Victor Babes” Clinical Hospital for Infectious Diseases in Timisoara. For each subject, baseline demographic data were collected, including sex and age, as well as anthropometric parameters obtained through bioimpedance analysis: body weight, height, and body mass index (BMI).

Lifestyle-related information included alcohol and tobacco use, as well as the level of physical activity (regular physical exercise versus sedentary behavior). Additionally, HIV-specific clinical parameters were recorded: nadir CD4 T-cell count, current CD4 T-cell count, current viral load, previous antiretroviral regimens (particularly those affecting bone metabolism), duration of HIV infection, and length of time on ART.

All participants underwent routine laboratory testing under fasting conditions, following established protocols. The biochemical markers assessed included total serum calcium (Ca), ionized calcium (the biologically active form), serum phosphate (P), 25-hydroxyvitamin D [25(OH)D], and magnesium (Mg).

Subsequently, a referral form was completed for each patient, directing them to the hospital’s bone density measurement department for DXA scanning. Pre-test conditions were strictly adhered to, ensuring participants had not used any radioactive substances or calcium-containing medications or supplements within the seven days prior the BMD assessment.

### 2.3. Bone Mineral Density Assessment

BMD was assessed using a DXA device—specifically, the General Electric Prodigy Primo (GE Medical Systems, Diegem, Belgium). Measurements were performed utilizing the local DXA protocol at three anatomical sites: total femur, lumbar vertebrae, and femoral neck. The DXA device was regularly calibrated according to daily quality control standards, and a special reference phantom was used to ensure measurement accuracy. The obtained values were expressed in grams per square centimeter (g/cm^2^).

Considering the age range of participants (18 to 80 years), both Z-scores (defined as an individual’s BMD compared to the average values of individuals of the same age, sex, race and body size) and T-scored (representing BMD compared to that of a young, healthy adult around 30 years old) were used for all analyses, following the guidelines of the World Health Organization (WHO) [28].

The interpretation of T-score values is as follows [29]: a T-score of >−1 indicates normal BMD, one between −1 and −2.5 signifies osteopenia, reflecting a lower-than-normal BMD that is not severe enough to be classified as osteoporosis, and a T-score of <−2.5 indicates osteoporosis, characterized by significantly reduced BMD and an increased risk of fractures.

The Z-score primarily assesses whether a patient’s BMD falls within the expected range for their age. Although less frequently used for diagnosis compared to the T-score, it provides valuable insights in specific contexts, such as assessing BMD in children, adolescents, premenopausal women, and young men. A score below −2 indicated that BMD is markedly below the average for the patient’s age group, which may warrant further investigation to uncover secondary factors contributing to the bone loss [29].

### 2.4. Variable Definition

For this study, the reference thresholds for biochemical markers associated with bone metabolism were established based on the standard values of our laboratory and by the current literature.

The serum concentration of 25-hydroxyvitamin D [25(OH)D] was categorized as deficient when it fell below 30 ng/mL, which is the widely accepted as the lower limit for adequate vitamin D status (reference range: 31–80 ng/mL) [30].

Total serum calcium was considered hypocalcemic if levels were below 8.4 mg/dL, in accordance with the reference range of the laboratory used in this study (8.4–10.2 mg/dL). Values below this threshold were interpreted as indicative of calcium deficiency. 

For ionized calcium, a threshold of 3.82 mg/dL was applied, below which hypocalcemia was considered present (reference range: 3.82–4.82 mg/dL). Although total serum calcium measurement is common due to its widespread availability, ionized calcium provides a direct measurement of the biologically active form of calcium and is less affected by serum protein level fluctuations [31,32]. Therefore, both parameters were assessed simultaneously to ensure a thorough evaluation of calcium status in patients.

Serum phosphorus levels below 2.2 mg/dL were classified as hypophosphatemia, as this is beneath the lower limit of laboratory’s reference range (2.2–4.7 mg/dL).

Magnesium levels were assessed using the laboratory’s standard reference interval, with a normal value defined as ranging from 1.6 to 2.6 mg/dL. Any value below 1.6 mg/dL was interpreted as hypomagnesemia.

### 2.5. Statistical Analysis

Statistical analysis was conducted using IBM SPSS software, version 26.0 (IBM Corp., Armonk, NY, USA). The dataset was initially evaluated for integrity, consistency, and the presence of outliers. The normality of continuous variable distributions was assessed using statistical methods and confirmed visually through histograms and Q–Q plots.

Results were summarized as mean ± SD for normally distributed variables and median with IQR for non-normal data. Categorical data were expressed as frequencies and percentages. Differences among three or more groups were analyzed using one-way Anova, with Levene’s test applied for variance homogeneity; if assumptions were violated, the Welch test was employed. Additional analyses included Chi-square or Fisher’s exact tests for categorical associations. A significance level of *p* < 0.05 was maintained throughout.

### 2.6. Ethical Consideration

The study protocol was thoroughly reviewed and approved by the Ethics Committee of the Clinical Hospital of Infectious Diseases and Pneumophysiology, “Victor Babes”, Timisoara, with approval number 3082 issued on 4 April 2023, ensuring adherence to ethical standards governing human research. Prior to participation, all individuals provided written informed consent, confirming their voluntary involvement, and understanding of the study’s objectives, procedures, and potential risk. The principles of confidentiality and the participants’ right to withdraw from the study at any point without repercussion were clearly communicated to all involved individuals.

## 3. Results

A total of 163 participants were enrolled in the study. Based on T-scores for BMD at the lumbar spine, 92 individuals (56.44%) exhibited low BMD, while 71 (43.56%) had a normal lumbar BMD. The majority of participants were male, accounting for 65.03% of the cohort. The mean age was 38,18 years (±9.87 SD). The baseline demographic and clinical characteristics of the population are summarized in Table 1.

Regarding BMD classification, 9.22% of individuals had T-scores below −2.5 indicating osteoporosis, 47.23% had scores between −2.5 and −1 suggestive of osteopenia, and 43.55% had T-scores above −1 reflecting normal bone density. Concerning vertebral fracture risk, those with T-scores above −1 were considered to have no lumbar fracture risk.

Assessment using Z-score showed that 84.05% of cases had values ≥ −2, indicating bone density within the expected range for age and sex, whereas 15.95% of individuals exhibited Z-scores below −2, indicative of significantly reduced bone density relative to their age-matched reference population. This may suggest the presence of secondary risk factors contributing to bone loss, warranting further investigation to identify underlying causes and implement appropriate preventive or therapeutic strategies.

Regarding the osteodensitometric assessment at the hip level (left femoral neck), according to the T-score, 2.45% of patients (*n* = 4) had values below −2.5, indicative of osteoporosis-level BMD. A further 39.27% (*n* = 64) fell within the −2.5 to −1 range, suggestive of osteopenia, while 58.28% (*n* = 95) had a T-score greater than −1, reflecting a normal bone density.

In the same region, 97.55% of patients (*n* = 159) exhibited a Z-score of ≥−2, indicating bone density within the expected range for their age and demographic profile; however, this does not exclude the potential risk of osteoporosis or fractures, especially when additional risk factors are present. Conversely, only 2.45% (*n* = 4) had a Z-score below −2, which could point to secondary causes of bone loss.

For the total femur, none of the patients exhibited a T-score below −2.5, indicating the absence of osteoporosis in this region. Approximately 28.83% (*n* = 47) had a T-score between −2.5 and −1, suggestive of osteopenia, while the majority, 71.17% (*n* = 116) demonstrated a T-score above −1, indicative of normal bone density.

According to the Z-score analysis, 99.38% of patients (*n* = 162) had values equal to or greater than −2 and only one patient (0.62%) recorded a Z-score below this threshold, highlighting the potential need for further investigations into the causes of decreased BMD in this area.

As presented in Table 2, statistical analysis did not reveal any significant association between bone mineral disorder presence and variables such as age, sex, or disease stage (HIV or AIDS) (*p* > 0.05). The mean duration since HIV diagnosis within the cohort was 11.46 ± 8.55 years. Although a downward trend in BMD across all three anatomical sites was observed with increasing time since diagnostic, this correlation did not reach statistical significance (all *p* > 0.05). The average duration of ART was 10.05 ± 6.9 years, and a similar non-significant inverse relationship was noted between treatment duration and BMD (all *p* > 0.05). The mean CD4 count was 509.8 ± 348.05 cells/µL with no significant correlation detected between CD4 levels and BMD in any assessed regions; (all *p* > 0.05). Additionally, CD4 nadir values showed no significant association with BMD across the evaluated anatomical areas. (*p* > 0.05).

An increased number of therapeutic regimens over time was significantly associated with lower BMD values at the hip (f = 2.06, *p* = 0.02) and femur (f = 2.35, *p* = 0.01), indicating a statistically significant cumulative treatment exposure effect. However, no significant association was observed regarding the spine region (f = 1.26, *p* = 0.24).

The mean BMD at the femoral neck was 0.86 ± 0.08 g/cm^2^ in patients with detectable viral load and 0.83 ± 0.25 g/cm^2^ in those with undetectable viremia, however this difference was not statistically significant (*p* = 0.25).

Conversely, a significant difference in total femur BMD was observed between patients with detectable and undetectable viral loads, with mean values of 0.98 ± 0.10 g/cm^2^ and 0.98 ± 0.08 g/cm^2^, respectively, (*p* = 0.03), though this difference is not deemed clinically relevant. Patients experiencing detectable viremia exhibited a higher prevalence of low BMD compared to those with suppressed viral loads, suggesting an association between ongoing viral activity and an increased risk of bone mineral density reduction in this region.

No significant association was found between lumbar spine BMD and ARN-HIV viral load, with mean values of 1.004 ± 0.09 g/cm^2^ in undetectable cases and 0.98 ± 0.10 g/cm^2^ in detectable cases (*p* > 0.05).

Body mass index (BMI) was not significantly linked with low BMD in any of the assessed regions (all *p* > 0.05); likewise, physical inactivity was not significantly associated with reduced BMD among the assessed patients (all *p* > 0.05), indicating these factors may not independently impact bone density in this cohort. Therefore, the results should be interpreted as indicating a lack of significant association rather than a definitive absence of effect.

Smoking was significantly associated with decreased BMD in the hip regions (*p* = 0.01) and lumbar spine (*p* = 0.001), but not at the femur (*p* > 0.05). Alcohol consumption corelated with decreased BMD at the femoral neck (*p* = 0.03), while no significant effect was observed at the total femur or lumbar spine (*p* > 0.05).

Regarding bone-related vitamins and minerals, total serum calcium and magnesium showed no significant associations with BMD across any regions (all *p* > 0.05). In contrast, low ionized calcium levels were significantly linked to reduced BMD in the lumbar spine (*p* = 0.01), serum phosphorus levels with the hip (*p* = 0.01), and vitamin D levels [25(OH)D] with the femoral neck (*p* = 0.01).

Regarding the multivariate logistic regression analysis, eleven ART drugs used among the study population exhibited significant associations with decreased BMD across all evaluated sites, suggesting a potential impact of these medications on skeletal health in HIV-infected patients.

Notably, within the NRTI class, Abacavir and TDF were indicated as statistically associated with decreased BMD in all the regions analyzed (*p* < 0.005 for each). These findings underscore the importance of careful monitoring of bone health in patients on these regimens.

Among PIs, Atazanavir was the only drug showing a significant association with reduced BMD across all evaluated regions (*p* < 0.005). This finding suggests that Atazanavir’s mechanism of action and its potential impact on bone health deserve further investigation and consideration in the management of antiretroviral therapy to mitigate its adverse impacts on skeletal integrity. 

Zidovudine was significantly linked to decreased BMD at the femur and lumbar spine (*p* < 0.005), while showing no signification at the hip (*p* = 0.057). Lopinavir and Stavudine showed a region-specific effect, with significant association only observed at the lumbar spine BMD, indicating localized impact on bone health.

Although two boosted drugs within the PI class (Atazanavir and Lopinavir) demonstrated an influence on bone density, Darunavir was not identified as a significant predictor of low BMD in any of the assessed regions, suggesting variability in their influence on bone health within the same drug class.

Regarding NRTI agents, four of them, Didanosine, Emtricitabine, Lamivudine, and TAF, did not show significant associations with decreased BMD (*p* > 0.005). These findings imply that, within the scope of this study, these antiretroviral agents do not exert a measurable impact on bone health in HIV-infected individuals.

## 4. Discussion

This study evaluated BMD in individuals PLWH and examined various factors potentially associated with decreased BMD across key skeletal regions. The findings revealed a considerable prevalence of low BMD, especially in the lumbar spine, underscoring the importance of routine bone health monitoring in this population.

With advances in ART and the consequent extension of life expectancy among PLWH, addressing the effects of aging has become a critical priority. In this context, reduced BMD emerges as a frequent complication. 

HIV infection directly contributes to bone loss through persistent systematic inflammation, accelerated bone turnover, and viral proteins that both stimulate osteoclastic activity and induce osteoblastic apoptosis [33]. Simultaneously, ART exerts a negative influence on bone health. The most significant decline in BMD typically occurs within the first few years after ART initiation [34], primarily due to immune reconstitution, increased bone resorption and the harmful effects of certain ART, notably TDF [35,36]. 

Meta-analyses substantiate that individuals undergoing ART have a twofold risk of developing osteopenia or osteoporosis compared to HIV-negative controls, accompanied with markedly lower lumbar and femoral BMD and a heightened incidence of fragility and vertebral fractures [20]. It has been emphasized that HIV-positive patients, matched by age and BMD to control groups, exhibit significantly lower lumbar BMD, underscoring the independent contributions of HIV infection and low body mass on bone deterioration [37]. The strong correlation between BMI and lumbar BMD highlights the importance of adequate nutrition, including optimal intake of calcium [38], phosphorus, magnesium, and vitamin D [39,40], to prevent bone demineralization. Existing data further reveal an increased prevalence of osteoporosis among HIV-infected individuals, especially in their fifth decade of life [41]. Consistent with these findings, in our study population, 56.44% of patients presented low BMD in the lumbar region and 39.26% displayed decreased density at the hip and femur, emphasizing the urgent need for regular BMD assessment within this demographic.

Recent research highlights the gut microbiota as a key factor in atherosclerosis development, influencing vascular inflammation through impacts on metabolism, immunity, and lipid processing [42], while lifestyle factors such as smoking and alcohol consumption significantly contribute to BMD reduction, with smoking associated with reduced BMD at the lumbar spine and hip, and alcohol negatively impacting the femoral neck, aligning with prior studies linking these modifiable risk factors to impaired bone metabolism and further bone loss in HIV-infected individuals [43]. 

Regarding biochemical parameters, total calcium and magnesium levels did not demonstrate a significant correlation with BMD in any anatomical region. In contrast, reduced ionized calcium levels were strongly associated with lower lumbar spine BMD (*p* = 0.001), while elevated serum phosphate levels were associated with reduced BMD at the hip (*p* = 0.01). Vitamin D deficiency also showed a significant negative association with BMD at the femoral neck (*p* = 0.01), suggesting that disturbances in mineral and vitamin homeostasis may contribute to skeletal demineralization within in this population [44]. 

Our findings indicated that the average time since HIV diagnosis among patients ranged from 1 to 34 years, with a mean of 11.46 ± 8.55 years. A decreased CD4 cell count was not identified to be a significant predictor for low BMD, in contrast to the result of other meta-analyses. Similarly, previous investigations have demonstrated no strong correlation between BMD and viral load, CD4 cell count, or therapy duration [45], whereas factors such as body weight and use of PI were identified as significant contributors. Although our data exhibited a notable association between viral load, the number of treatment regimens over time, and low BMD risk, these variables did not attain significance across all assessed regions. The multifactorial nature of these associations, as reported in other studies, suggests that low CD4 counts and the length of HIV infection may influence bone health through direct viral effects on bone metabolism, though our findings did not substantiate this hypothesis. Additionally, the use of certain ART, such as PIs or NRTIs, coupled with weight fluctuations, may further impact BMD reduction.

A comparable study conducted in Romania reported a relatively high prevalence of osteopenia (15%) at both the hip and lumbar spine and osteoporosis (5% at these sites) despite the relatively young age of the study population. Furthermore, other research focusing on Romanian PLWH indicated that osteoporosis and osteopenia were more frequently observed in the hip among females, whereas, in males, the lumbar spine was more commonly affected [25].

While prior research has suggested that Abacavir does not markedly affect BMD, our results demonstrate a notable decline in BMD among patients undergoing Abacavir-based treatment regimens [46]. Analysis of ART indicated that Abacavir, TDF [47,48], and Atazanavir were significantly linked to reduced BMD across all evaluated anatomical regions. Zidovudine [39] was associated with a lower BMD at the femur and lumbar spine but showed no effect at the hip. Lopinavir showed a significant association only at the lumbar spine, whereas Darunavir was not statistically related to BMD. Additionally, half of the examined NRTIs (Didanosine, Emtricitabine, Lamivudine and TAF) that are still widely used in HIV management did not demonstrate any influence on BMD. These results suggest a drug-specific effect of ART on bone metabolism, emphasizing the need for individualized treatment approaches and regular bone density evaluation, particularly in patients undergoing long-term therapy. 

### Further Implications

The present study opens significant avenues for both clinical practice and future research. Firstly, our findings underscore the need for larger, long-term cohort studies to validate these results and to better understand the complex interaction between biochemical, therapeutic, biological, and risk factors affecting BMD in individuals living with HIV. Such research is crucial for developing personalized prevention and treatment strategies aimed at maintaining bone mass, reducing osteoporotic complications, and establishing effective preventive measures. Furthermore, the varying prevalence of low BMD among different HIV-positive subgroups underscores the importance of age and risk-tailored screening protocols. Evaluating the benefits of routine bone density assessments in patients under 50 could facilitate early detection of osteopenia and osteoporosis, enabling timely intervention and preventive care.

## 5. Conclusions

This study underscores significant considerations for practice and future research in bone health management among HIV-infected individuals. While avoiding smoking and alcohol may reduce the risk of BMD loss, it does not necessarily lead to increased bone density. Conversely, deficiencies in phosphorus and vitamin D are associated with reduced BMD in the femoral neck and femur areas. Additionally, ART regimens including Abacavir, TDF, and Atazanavir show a consistent link to lower BMD at all evaluated sites. These findings emphasize the multifactorial nature of bone health challenges in this population and advocate for comprehensive prevention and monitoring strategies to maintain bone integrity.

## Figures and Tables

**Table 1:** Main characteristics of patients.

Variables	*Total Population*	*Lumbar*		*Total Femur* *Left Femoral Neck*	
*Categorical Variables*		*Low* *BMP Group*	*Normal* *BMP Group*	*Low* *BMP Group*	*Normal* *BMP Group*
	*(n = 163)*	*(n = 92)*	*(n = 71)*	*(n = 64)*	*(n = 99)*
Male	106 (65.03)	62 (67.39)	44 (61.97)	45 (70.31)	61 (61.61)
Female	57 (34.97)	30 (32.6)	27 (38.02)	19 (29.68)	38 (38.38)
Smoking	80 (49.07)	54 (58.69)	24 (33.80)	39 (60.93)	41 (41.41)
Alcohol	20 (12.26)	12 (13.04%)	8 (11.26%)	11 (17.18)	9 (9.09)
NO Physical activity	79 (48.46)	47 (51.08%)	32 (45.07%)	38 (59.37)	41 (41.41)
Detectable viral load	56 (34.35)	33 (35.86%)	23 (32.39%)	22 (34.37)	34 (34.34)
AIDS	117 (71.77)	65 (70.65%)	52 (73.23%)	49 (76.56)	68 (38.38)
***Previous treatment regimens***					
***NRTI-regiment***					
Abacavir (ABC)	85 (52,14)	61 (66.30)	24 (33.80)	39 (60.93)	46 (46.46)
Didanozine (ddI)	53 (32.51)	24 (26.08)	29 (40.84)	26 (40.62)	27 (27.27)
Emtricitabine (FTC)	146 (89.57)	83 (90.21)	63 (88.73)	59 (92.18)	87 (87.87)
Lamivudine (3TC)	123 (75.4)	69 (75.00)	54 (76.05)	50 (78.12)	73 (73.73)
Stavudine (d4T)	90 (55.21%)	56 (60.86)	34 (47.88)	39 (60.93)	51 (51.51)
Tenofovir alafemanide (TAF)	129 (79.14)	71 (77.17)	58 (81.69)	52 (81.25)	76 (76.76)
Tenofovir disoproxil fumarate (TDF)	116 (71.16%)	72 (78.26)	44 (61.97)	54 (84.37)	62 (2.62)
Zidovudine (ZDV)	55 (33.74%)	34 (36.95)	21 (29.57)	27 (42.18)	28 (28.28)
*Boosted protease inhibitors (with ritonavir or cobicistat)*					
Atazanavir (ATV)	72 (44.17)	58 (63.04)	14 (19.71)	36 (56.25)	36 (36.36)
Darunavir (DRV)	100 (61.34)	54 (58.69)	46 (64.78)	40 (62.5)	60 (60.60)
Lopinavir (LPV)	57 (34.96)	45 (48.91)	12 (16.90)	27 (42.18)	30 (30.30)
***Continuous variables***					
Age (year)	38.18 ± 9.87	39.1 ± 11.02	36.98 ± 7.97	40.65 ± 10.77	36.58 ± 8.87
Female (age)	38.05 ± 7.02	39.16 ± 8.74	36.81 ± 4.03	38.31 ± 7.75	37.92 ± 6.62
Male (age)	38.25 ± 11.10	39.08 ± 11.97	37.09 ± 9.62	41.64 ± 11.68	35.75 ± 9.94
Time since HIV diagnostic (year)	11.46 ± 8.55	11.46 ± 8.33	11.46 ± 8.82	13.17 ± 9.30	10.36 ± 7.82
Treatment duration (year)	10.05 ± 6.90	10.25 ± 7.07	9.8 ± 6.67	11.57 ± 7.75	9.07 ± 6.09
Previous treatment regimens (number)	3.37 ± 2.76	3.44 ± 2.82	3.28 ± 2.68	4.04 ± 2.98	2.94 ± 2.52
Cd4 nadir (cells/μL)	256.36 ± 238.41	226.44 ± 200.83	295.12 ± 274.88	240. 85 ± 207.07	269.09 ± 258.56
Cd4 cells (cellsu/μL)	509.80 ± 348.05	485.68 ± 317.01	541.07 ± 382.31	465.98 ± 333.07	538.14 ± 354.53
Serum Ca (mg/dL)	9.03 ± 0.64	9.08 ± 0.6	9.13 ± 0.59	9.03 ± 0.6	9.15 ± 0.58
Ionized Ca (mg/dL)	3.84 ± 0.37	3.82 ± 0.37	3.85 ± 0.37	3.82 ± 0.40	3.85 ± 0.35
P (mg/dL)	3.04 ± 0.6	2.96 ± 0.56	3.18 ± 0.6	2.97 ± 0.65	3.10 ± 0.53
Mg (mg/dL)	1.95 ± 0.2	1.94 ± 0.2	1.95 ± 0.19	1.92 ± 0.18	1.95 ± 0.19
Vitamin D (ng/mL)	22.46 ± 11.67	23.09 ± 10.86	22.59 ± 13.44	23.35 ± 11.11	22.56 ± 12.61
BMI (kg/m^2^)	23.63 ± 4.92	23.55 ± 4.99	23.57 ± 4.82	22.2 ± 3.79	24.57 ± 5.37
Femoral Neck T	−0.761 ± 0.87	−1.046 ± 0.836	−0.39 ± 0.781	−1.58 ± 0.54	−0.23 ± 0.59
Femoral Neck Z	−0.38 ± 0.85	−0.62 ± 0.834	−0.07 ± 0.753	−1.13 ± 0.52	0.10 ± 0.63
Femoral Neck BMD (g/cm^2^)	0.792 ± 0.167	0.769 ± 0.110	−0.823 ± 0.215	0.70 ± 0.07	0.85 ± 0.18
Total hip T-score	−0.53 ± 0.76	−0.76 ± 0.72	−0.23 ± 0.7	1.19 ± 0.47	−0.09 ± 0.56
Total hip Z-score	−0.36 ± 0.82	−0.53 ± 0.75	−0.1 ± 0.71	−0.97 ± 0.48	0.06 ± 0.61
Total HIP BMD (g/cm^2^)	0.924 ± 0.111	0.895 ± 0.10	0.961 ± 0.108	0.83 ± 0.07	0.98 ± 0.08
Lumbar T-score	−1.16 ± 0.97	−1.85 ± 0.56	−0.35 ± 0.98	−1.78 ± 1.12	−0.84 ± 0.81
Lumbar Z-score	−0.96 ±0.96	−1.6 ± 0.63	−0.12 ± 0.61	−1.44 ± 0.96	−0.66 ± 0.79
Lumbar BMD (g/cm^2^)	0.952 ± 0.133	0.873 ± 0.065	1.054 ± 0.127	0.89 ± 0.16	0.98 ± 0.09

Data are indicated as mean ± SD, median (IQR), or N (%). Abbreviations: HIV, human immunodeficiency virus; BMD, bone mineral density; AIDS, acquired immunodeficiency syndrome; CD4, cluster of differentiation 4; Ca, calcium; P, phosphorus; Mg, magnesium; BMI, body mass index.

**Table 2:** Correlation coefficient of variables and BMD of different regions.

	*Femoral Neck BMD*		*Total Hip—BMD*		*LUMBAR BMD*	
	*Correlation Coefficient*	*p-Value*	*Correlation Coefficient*	*p-Value*	*Correlation Coefficient*	*p-Value*
Age (year)	1.24	0.18	0.97	0.52	0.98	0.50
Sex	0.04	0.39	0.58	0.29	1.21	0.18
AIDS	0.78	0.37	0.41	0.52	0.82	0.36
Time since HIV diagnostic (year)	1.07	0.37	1.20	0.23	1.03	0.43
Treatment duration (year)	0.32	0.99	0.68	0.87	0.6	0.93
Previous treatment regimens (number)	2.06	0.02 *	2.35	0.01 *	1.26	0.24
CD4 cells nadir	1000	0.51	1.08	0.39	0.66	0.95
CD4 cells	1.15	0.43	0.65	0.86	0.51	0.95
Detectable viral load	1.63	0.25	3.64	0.03 *	1.44	0.32
BMI	0.71	0.93	0.82	0.80	1.14	0.29
Smoking	0.13	0.01 *	0.52	0.09	2.68	0.001 *
Alcohol	0.87	0.03 *	0.004	0.07	2.80	0.08
NO Physical activity	0.27	0.75	2.56	0.08	0.62	0.53
Ionized Ca	1.24	0.16	1.15	0.26	1.68	0.01 *
Serum Ca	0.2	0.15	1.03	0.3	0.13	0.7
P	1.84	0.01 *	1.26	0.19	0.67	0.87
Mg	0.92	0.63	0.79	0.83	1.38	0.07
Vitamin D	1.32	0.20	2.23	0.01 *	0.97	0.55
***Previous treatment regimens***						
***NRTI—regiment***						
ABC	0.53	0.04 *	0.01	0.05 *	0.5	0.01 *
ddI	0.06	0.8	1.36	0.24	0.2	0.65
FTC	0.35	0.55	3.31	0.07	0.39	0.53
3TC	0.03	0.84	0.11	0.73	0.11	0.74
d4T	0.36	0.49	0.88	0.48	0.08	0.02 *
TAF	5.52	0.43	0.75	0.6	0.34	0.89
TDF	0.13	0.001 *	1.29	0.01 *	0.03	0.05 *
ZDV	0.65	0.057	0.58	0.02 *	0.09	0.02 *
***Boosted protease inhibitors***						
ATV	0.01	0.002 *	0.044	0.01 *	3.74	0.001 *
DRV	0.04	0.84	0.09	0.68	0.17	0.64
LPV	0.09	0.17	0.14	0.07	2.82	0.002 *

Abbreviations: HIV, human immunodeficiency virus; BMD, bone mineral density; AIDS, acquired immunodeficiency syndrome; CD4, cluster of differentiation 4; BMI, body mass index; Ca, calcium; ddI, Didanozina; FTC, Emtricitabina; 3TC, Lamivudina; P, phosphorus; Mg, magnesium; ABC, Abacavir; TDF, Tenofovir Disoproxil fumarate; ZDV, Zidovudine; d4T, Stavudine; ATV, Atazanavir; DRV, Darunavir; LPV, Lopinavir, * *p* < 0.05.

## Data Availability

The available data can be provided upon request to the corresponding author (due to internal regulations of the hospital—Regulation UE nr. 679 from 2016 regarding protection of personal data).

## Abbreviations

The following abbreviations are used in this manuscript:

ABC	Abacavir
AIDS	Acquired Immunodeficiency Syndrome
ART	Antiretroviral Therapy
ATV	Atazanavir
BMD	Bone Mineral Density
BMI	Body Mass Index
Ca	Calcium
CD4	Cluster of Differentiation 4
DRV	Darunavir
DXA	Dual-Energy X-ray Absorptiometry
HAART	Highly Active Antiretroviral Therapy
HIV	Human Immunodeficiency Virus
LPV	Lopinavir
Mg	Magnesium
P	Phosphate
PLWH	People living with HIV
RNA	Ribonucleic Acid
d4T	Stavudine
TAF	Tenofovir alafenamide
TDF	Tenofovir Disoproxil fumarate
WHO	World Health Organization
ZDV	Zidovudine
